# Correction: Xu et al. MC-ASFF-ShipYOLO: Improved Algorithm for Small-Target and Multi-Scale Ship Detection for Synthetic Aperture Radar (SAR) Images. *Sensors* 2025, *25*, 2940

**DOI:** 10.3390/s26020696

**Published:** 2026-01-21

**Authors:** Yubin Xu, Haiyan Pan, Lingqun Wang, Ran Zou

**Affiliations:** School of Information Science, Shanghai Ocean University, Shanghai 201306, China; 2226234@st.shou.edu.cn (Y.X.); hy-pan@shou.edu.cn (H.P.); 2152730@st.shou.edu.cn (R.Z.)

## Legends of Figures 1 and 3

In the original publication [1], the source attribution for Figures 1 and 3 were inadvertently omitted. The corrected Figures 1 and 3 appears below. 

**Figure 1.** (**a**) Ship targets in complex inland river environments; (**b**) scene showing densely distributed small ship targets; (**c**) examples of multi-scale ship targets after zero-padding processing of the SSDD dataset. Note that (**a**,**b**) are from the HRSID dataset. Red bounding boxes highlight real ships.**Figure 3.** Overall framework of the MC-ASFF-ShipYOLO model based on YOLO11 improvements, with improved modules highlighted in red boxes. The foundational YOLO11 network structure is independently drawn based on the Ultralytics configuration files [43].

## Missing Citation

In the original publication, ref. [43] was not cited. The citation has now been inserted in Section 1, Paragraph 6 and should read as follows:

To overcome these limitations, we propose MC-ASFF-ShipYOLO (Monte Carlo Attention—Adaptively Spatial Feature Fusion—ShipYOLO), an improved single-stage detector based on YOLO11 [43,44]. Our main contributions are as follows.

In addition, the citation has been inserted in Section 2.4, Paragraph 1 and should read as follows:

MC-ASFF-ShipYOLO is an improved model based on the YOLO11 object detection network. As a YOLO series model released on 30 September 2024 [43], YOLO11 achieves significant improvements in feature extraction capability and small object detection performance compared to previous versions, with the advantages of higher precision, fewer parameters, and faster inference speed.

The citation has also been inserted in Section 3.1, Paragraph 1 and should read as follows:

In this section, we chose the two-stage detectors, alongside the high-efficiency neural network model EfficientNet, and incorporated various editions of the YOLO series (such as YOLOv8 [52], YOLOv9 [53], YOLOv10 [54], YOLO11 [43], and YOLOv12 [55]) to conduct comparative experiments. Table 3 provides a detailed comparison of the experimental outcomes.

In the original publication, ref. [45] was not cited. The citation has now been inserted in Section 2.1, Paragraph 1 and should read as follows:

This study employs a hybrid dataset, constructed from HRSID and SSDD, as the basis for model training and performance evaluation. Details of the hybrid dataset can be found in the Supplementary Materials. Released in 2020, HRSID [45] is a high-resolution SAR image dataset designed for ship detection and segmentation tasks.

In the original publication, ref. [46] was not cited. The citation has now been inserted in Table 2, as shown below:**Models****Backbone****Batch** **Size****lr0****lrf****NMS**Faster R-CNNR50160.010.00010.6R101120.010.00010.7Cascade R-CNNR50160.010.00010.6R101160.010.00010.7EfficientNet [46]Eff-b360.010.0001-

In the original publication, refs. [52–55] were not cited. The citation [52] has now been inserted in Section 3, Paragraph 1 and should read as follows:

This section provides a detailed evaluation of the MC-ASFF-ShipYOLO model’s performance on the constructed hybrid SAR ship dataset and systematically compares it with classical object detection networks. We conduct a comprehensive assessment of the improved model’s effectiveness in SAR ship detection tasks through both quantitative metrics and qualitative analysis. YOLO11 is an improved version of YOLOv8 [52].

The citations [52–55] have now been inserted in Section 3.1, Paragraph 1 and should read:

In this section, we chose the two-stage detectors, alongside the high-efficiency neural network model EfficientNet, and incorporated various editions of the YOLO series (such as YOLOv8 [52], YOLOv9 [53], YOLOv10 [54], YOLO11 [43], and YOLOv12 [55]) to conduct comparative experiments. Table 3 provides a detailed comparison of the experimental outcomes.

## Text Correction

In the original publication, the model was referred to using non-standard terminology. The name “YOLO12” has been updated to the standardized designation “YOLOv12” for technical accuracy. A correction has been made to Results, Section 3.1, Paragraph 1:

In this section, we chose the two-stage detectors, alongside the high-efficiency neural network model EfficientNet, and incorporated various editions of the YOLO series (such as YOLOv8 [52], YOLOv9 [53], YOLOv10 [54], YOLO11 [43], and YOLOv12 [55]) to conduct comparative experiments. Table 3 provides a detailed comparison of the experimental outcomes.

A correction has also been made to Table 3:
**Models****Backbone** **or Size**P**/%**R**/%**$AP_{50}$**/%**AP**/%****Params (M)****FPS (img/s)**YOLOv8n90.8582.8390.6764.053.01666.67s90.7484.7591.8165.2411.13370.37YOLOv9t91.1183.8291.5565.761.97555.57s90.9084.9491.9767.537.17333.33YOLOv10n88.9081.5590.1165.202.70666.67s91.0983.4091.7666.778.04384.62YOLO11n90.4181.5689.2063.922.58555.57s92.4884.2192.7867.409.41370.37YOLOv12n90.9381.0890.4366.042.56416.67s91.3982.2191.5966.249.23243.90EfficientNetEff-b374.9189.2988.0061.3018.34306.30Faster R-CNNR5084.1882.8783.4060.8041.35308.10R10184.1883.0883.2060.5060.34324.00Cascade R-CNNR5084.5183.9884.5063.1069.15317.50R10184.9784.1383.8062.8088.14317.10MS-ASFF- ShipYOLO (Ours)-93.8786.8494.5670.4460.28232.56

## Supplementary Materials

A direct mention of the Supplementary Materials has been added to Section 2.1, Paragraph 1 and should read as follows:

This study employs a hybrid dataset, constructed from HRSID and SSDD, as the basis for model training and performance evaluation. Details of the hybrid dataset can be found in the Supplementary Materials. Released in 2020, HRSID [45] is a high-resolution SAR image dataset designed for ship detection and segmentation tasks.

**Supplementary Materials:** The raw data supporting the findings of this study are available at the following link: https://pan.baidu.com/s/15XwGxaZddf94N_V_MkCqgw?pwd=kzpk (accessed on 30 December 2025).

## References

43. Jocher, G.; Qiu, J. Ultralytics YOLO11 (Version 11.0.0). 2024. Available online: https://github.com/ultralytics/ultralytics (accessed on 30 December 2025).45. Wei, S.; Zeng, X.; Qu, Q.; Wang, M.; Su, H.; Shi, J. HRSID: A high-resolution SAR images dataset for ship detection and instance segmentation. *IEEE Access* **2020**, *8*, 120234–120254.46. Tan, M.; Le, Q. Efficientnet: Rethinking model scaling for convolutional neural networks. In Proceedings of the International Conference on Machine Learning, Long Beach, CA, USA, 9–15 June 2019; pp. 6105–6114.52. Jocher, G.; Chaurasia, A.; Qiu, J. Ultralytics YOLOv8 (Version 8.0.0). 2023. Available online: https://github.com/ultralytics/ultralytics (accessed on 30 December 2025).53. Wang, C.-Y.; Yeh, I.-H.; Mark Liao, H.-Y. Yolov9: Learning what you want to learn using programmable gradient information. In *European Conference on Computer Vision*; Springer Nature: Cham, Switzerland, 2024; pp. 1–21.54. Wang, A.; Chen, H.; Liu, L.; Chen, K.; Lin, Z.; Han, J. Yolov10: Real-time end-to-end object detection. *Adv. Neural Inf. Process. Syst.*
**2024**, *37*, 107984–108011.55. Tian, Y.; Ye, Q.; Doermann, D. Yolov12: Attention-centric real-time object detectors. *arXiv* **2025**, arXiv:2502.12524.

With this correction, the order of some references has been adjusted accordingly.

The authors state that the scientific conclusions are unaffected. This correction was approved by the Academic Editor. The original publication has also been updated.

## References

[1] Xu Y., Pan H., Wang L., Zou R. (2025). MC-ASFF-ShipYOLO: Improved Algorithm for Small-Target and Multi-Scale Ship Detection for Synthetic Aperture Radar (SAR) Images. Sensors.

